# Optimizing patient outcomes: the impact of multimodal preemptive analgesia in video-assisted thoracoscopic lobectomy

**DOI:** 10.1093/icvts/ivae096

**Published:** 2024-05-16

**Authors:** Bing Li, Yu Chen, Rong Ma

**Affiliations:** Department of Anesthesiology, Jiangsu Province Hospital and First Affiliated Hospital with Nanjing Medical University, Nanjing City, China; Department of Anesthesiology, Jiangsu Province Hospital and First Affiliated Hospital with Nanjing Medical University, Nanjing City, China; Department of Anesthesiology, Jiangsu Province Hospital and First Affiliated Hospital with Nanjing Medical University, Nanjing City, China

**Keywords:** Ultrasound-guided thoracic paravertebral block, Intravenous analgesia, Enhanced recovery after surgery, Video-assisted thoracoscopic lobectomy

## Abstract

**OBJECTIVES:**

The aim of this study was to evaluate the efficacy of a multimodal preemptive analgesia management approach, specifically incorporating ultrasound-guided thoracic paravertebral block (UG-TPVB) in conjunction with intravenous analgesia, after video-assisted thoracoscopic (VATS) lobectomy under the guidance of enhanced recovery after surgery.

**METHODS:**

A total of 690 patients who underwent VATS lobectomy between October 2021 and March 2022 were divided into the UG-TPVB group (group T, *n* = 345) and the control group (group C, *n* = 345). Patients in group T received UG-TPVB prior to the induction of general anaesthesia, while group C did not undergo nerve block. A comparison was conducted between the 2 groups regarding various indicators, including postoperative sedation, static/dynamic numeric rating scale scores, intraoperative fentanyl consumption, duration of mechanical ventilation/anaesthesia recovery/hospitalization, postoperative complications and other relevant factors.

**RESULTS:**

The static/dynamic numeric rating scale scores of group T were lower than those of group C after surgery. Intraoperative fentanyl consumption in group T (0.384 ± 0.095 mg) was lower than that in group C (0.465 ± 0.053 mg). The duration of mechanical ventilation, anaesthesia recovery and hospitalization were significantly shorter in group T compared to group C. Patient satisfaction rate in group T (70.1%) was higher than that in group C (53.6%). All differences were statistically significant (*P* < 0.05).

**CONCLUSIONS:**

The multimodal preemptive analgesia management strategy effectively reduces postoperative pain, decreases opioid consumption and promotes faster recovery in patients undergoing VATS lobectomy.

## Graphical Abstract

**Figure:**
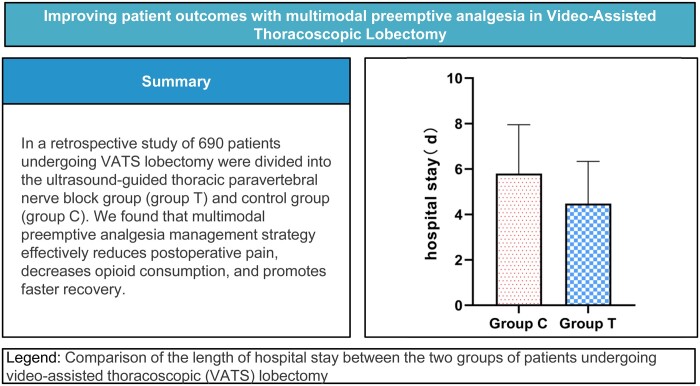

## INTRODUCTION

VATS lobectomy is a frequently employed therapeutic approach for individuals diagnosed with early or locally advanced non-small-cell lung cancer due to its reduced invasiveness, lower postoperative complication rates and cost-effectiveness compared to traditional thoracotomy [1–6]. However, localization of pulmonary lesions through CT-guided（computed tomography） needle puncture and chest drain placement can result in significant postoperative pain, hindering rapid recovery [4]. In recent years, the enhanced recovery after surgery (ERAS) concept has gained popularity, aiming to optimize clinical pathways through multidisciplinary collaboration and reduce perioperative complications while promoting early patient recovery [7–9]. Adequate perioperative analgesia is a critical component of the ERAS philosophy, which aims to improve patient quality of life and satisfaction by implementing a multimodal, preventive, low-opioid analgesic approach [10]. This study compares the clinical effects of multimodal analgesia in combination with nerve blocks and intravenous analgesia alone, providing a reference for selecting optimized analgesia following thoracoscopic lobectomy.

## MATERIALS AND METHODS

### Ethical statement

This study was retrospective and approved by the Ethics Committee of the First Affiliated Hospital with Nanjing Medical University (approval number: 2022-SR-543).

### Patient selection

A total of 690 patients undergoing elective thoracoscopic lobectomy between October 2021 and March 2022 were enrolled and divided into the ultrasound-guided thoracic paravertebral nerve block group (group T) and control group (group C).

Inclusion criteria were as follows: (i) age ≥18 years, no gender limitation, (ii) body mass index between 18 and 32 kg/m^2^ and (iii) American Society of Anesthesiologists class I and II. Exclusion criteria included: (i) abnormal coagulation function, (ii) anaesthetic allergies, (iii) severe cardiopulmonary insufficiency, (iv) significant liver or kidney insufficiency and (v) long-term opioid use. Elimination criteria included: (i) block failure, (ii) change of surgical method during surgery (such as conversion to open surgery) and (iii) delayed extubation due to bleeding or other reasons.

### Interventions

All patients were routinely fasted, and no preoperative medication was given. Upon entering the operating room, intravenous access was established, and standard monitoring, including three-lead continuous electrocardiogram (ECG), invasive blood pressure and pulse oximetry, was applied. Group T patients received an intravenous injection of midazolam 1 mg and fentanyl 0.05 mg, followed by ultrasound-guided thoracic paravertebral block (UG-TPVB). Group C patients were administered endotracheal intubation under general anaesthesia directly. Anaesthesia induction, maintenance and postoperative intravenous analgesia plans were identical for both groups. Patients received a postoperative controlled intravenous analgesia pump containing 30 mg oxycodone, 16 mg ondansetron, 3 mg droperidol, diluted to 150 ml with normal saline. The initial dose was 0 ml, with a background continuous dose of 1 ml, single pressing dose of 6 ml and a locking time of 15 min. This continued until 48 h postoperation, when the analgesia pump drug infusion was nearly complete and replaced by the postoperative follow-up personnel. All patients received a two-port VATS approach, which was 1 camera port of 1 cm at the seventh intercostal incision in the midaxillary line and 1 utility incision of 3–5 cm at the fourth intercostal incision between the anterior axillary line and the midclavicular line. Following the surgical procedure, individuals were relocated to the postanaesthesia care unit. The endotracheal tube was extracted once the extubation criteria were satisfied, and patients were subsequently discharged from the postanaesthesia care unit upon fulfilling the discharge criteria and subsequently returned to their respective wards.

#### Ultrasound-guided thoracic paravertebral block

Patients were placed in the lateral decubitus position (affected side on top, consistent with the surgical position). Routine skin disinfection was performed, and the T4 and T7 paravertebral spaces were located using a low-frequency convex array ultrasound probe (FUJIFILM Sonosite, Inc., Bothell, WA, USA). The puncture needle for nerve block was injected into the paravertebral space through the short-axis plane of the spine. If no air or blood was drawn, 15 and 20 ml of local anaesthetic mixture (including 0.375% ropivacaine and 0.125 mg/ml dexamethasone) were injected into the T4 and T7 paravertebral spaces, respectively. After the procedure, patients were assisted in transitioning from the lateral decubitus position to the supine position. The block plane was determined after a 20-min observation period. If skin pain and temperature sensation disappeared on the affected side and the block range covered at least the T4–T9 sensory plane segment, the block was considered successful and effective. An anaesthesiologist experienced in nerve block performed the UG-TPVB throughout the trial to ensure consistency.

## OUTCOMES

### Primary outcomes

#### Patient analgesia evaluation

Static/dynamic numeric rating scale (NRS) scores (0 points painless; 1–3 points mild pain, not affecting sleep; 4–6 points moderate pain, affecting sleep but still able to sleep; 7–10 points severe pain, preventing sleep) collected at 4, 12, 24 and 48 h postoperation. The dynamic NRS score was taken after patients performed a deep breath. Intraoperative fentanyl consumption was recorded, and the incidence of rescue analgesia was analysed.

#### Accelerated postoperative recovery assessment

Operation duration (from the beginning of the operation to the end), mechanical ventilation duration (from the end of anaesthesia to the complete withdrawal of the endotracheal tube), anaesthesia recovery duration (from the end of anaesthesia to departure from the operating room) and hospitalization duration (from the end of the operation to the day of discharge) were recorded.

### Secondary outcomes

Sedation scores (0 points sober; 1 point slightly drowsy but easy to wake up; 2 points frequently drowsy, able to wake up but unable to remain awake, potentially falling asleep during conversation; 3 points difficult to wake) were recorded at 24 and 48 h postoperation. Adverse reactions (nausea, vomiting, dizziness, pruritus, urinary retention, etc.) were observed during the same time intervals. The study documented the total number of effective compressions of the analgesic pump within 48 h postoperation and concurrently evaluated patient satisfaction with comfort levels at 48 h postoperation using a scoring system ranging from 0 (indicating dissatisfaction) to 2 (indicating high satisfaction).

### Statistical analysis

Statistical analyses were executed employing IBM SPSS Statistics version 25.0 software. Continuous variables exhibiting normal distribution were denoted as the mean ± standard deviation (*x̅* ± s), and the comparisons between the 2 groups were conducted utilizing independent samples t-tests. In instances where the data followed a skewed distribution, the values were represented as the median along with the interquartile range [*M* (P25, P75)], and non-parametric Mann–Whitney *U* tests were employed for group comparisons. Categorical variables were described as proportions (percentages) and analysed using Pearson's chi-square tests or Fisher's exact tests, as appropriate. A *P*-value of <0.05 (*P* < 0.05) was deemed to indicate statistical significance, thereby suggesting the presence of a meaningful difference or association between the groups under investigation.

## RESULTS

### General data

Demographic data (age and sex), body mass index and duration of surgery are presented in Table 1. There were no differences in the baseline characteristics between the groups(*P* > 0.05).

**Table 1:** Patients' baseline characteristics between 2 groups

Variable	Group T (*n* = 345)	Group C (*n* = 345)	Statistics	*P*-value
Age (years)	56.34 ± 11.52	56.29 ± 11.37	0.063	0.950
Sex, *n* (%)				
Male	162 (47.0%)	129 (37.4%)	6.472	0.011
Female	183 (53.0%)	216 (62.6%)		
BMI (kg/m^2^)	23.80 ± 2.84	23.66 ± 2.64	0.688	0.492
Duration of surgery (min)	103.03 ± 44.59	99.21 ± 41.19	1.167	0.244

Values are represented as mean ± SD, median (P25, P75) or number (%).

BMI: body mass index; SD: standard deviation.

### Primary outcomes

**Figure 1:**
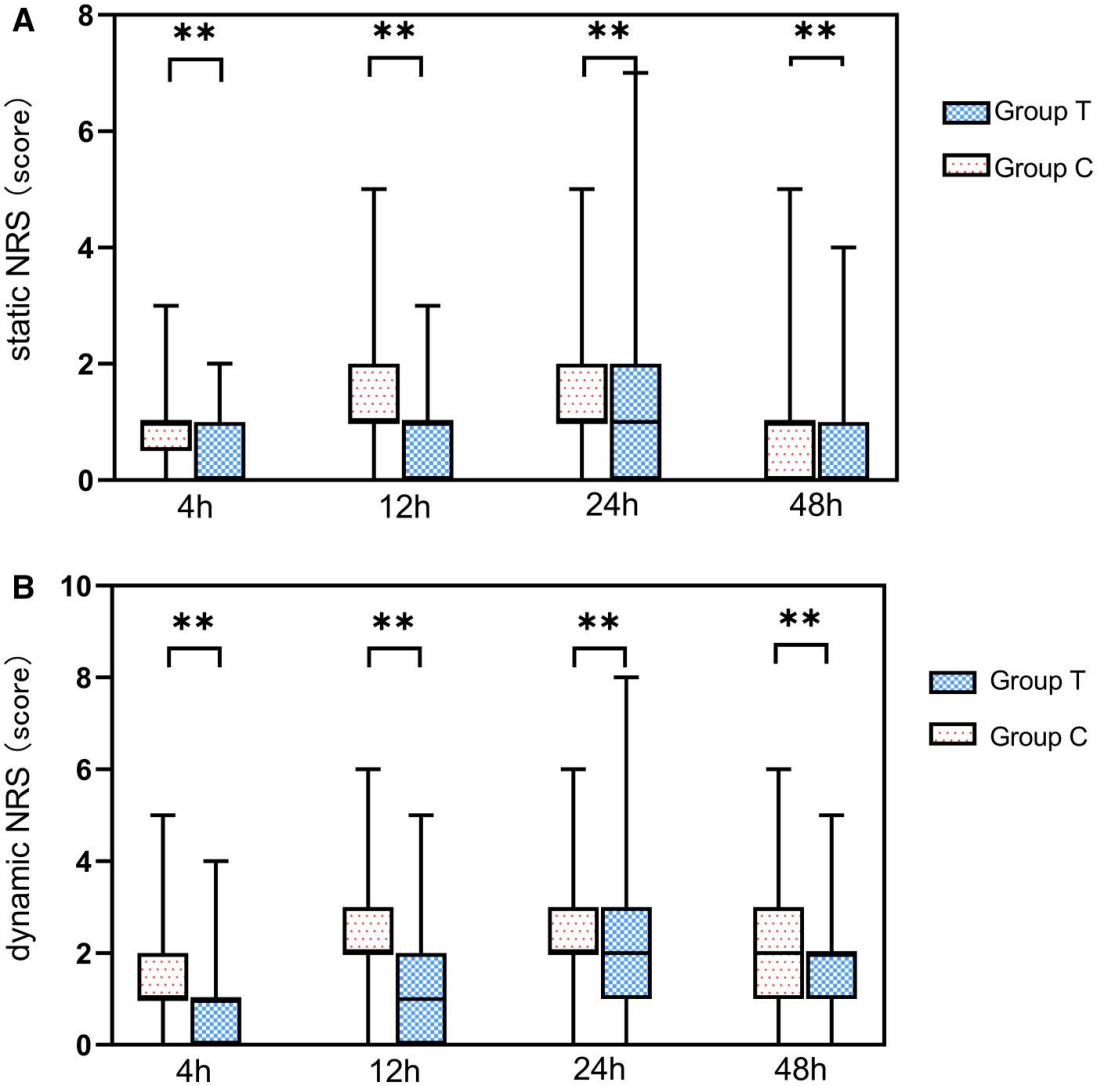
Comparison of static/dynamic NRS scores (**A** and **B**, respectively) in group T and group C at 4, 12, 24 and 48 h post-surgery. Group C underwent general anaesthesia alone. In contrast, group T received both ultrasound-guided thoracic paravertebral nerve block and general anaesthesia. Data are expressed as median (P25, P75), **P < 0.01.

The NRS scores are presented in 2 separate sections: (i) static and (ii) dynamic. The NRS scores for group T were, on average, lower than those for group C across various time points post-surgery (*P* < 0.05) (Table 2, Fig. 1). However, individual patient in group T exhibited a higher NRS score than those in group C at the 24-h postoperative time point (Fig. 1).

**Table 2:** Postoperative numerical rating scale andintraoperative fentanyl consumption

Primary outcome variable	Group T (*n* = 345)	Group C (*n* = 345)	Statistics	*P*-value
Static NRS (score)				
4 h	0 (0, 1)	1 (0, 1)	12.356	<0.001
12 h	1 (0, 1)	1 (1, 2)	10.152	<0.001
24 h	1 (0, 2)	1 (1, 2)	3.064	0.002
48 h	0 (0, 1)	1 (0, 1)	4.272	<0.001
Dynamic NRS (score)				
4 h	1 (0, 1)	1 (1, 2)	13.107	<0.001
12 h	1 (0, 2)	2 (2, 3)	10.877	<0.001
24 h	2 (1, 3)	2 (2, 3)	3.787	<0.001
48 h	2 (1, 2)	2 (1, 3)	4.597	<0.001
Fentanyl consumption (mg)	0.384 ± 0.095	0.465 ± 0.053	13.911	<0.001

Values are represented as mean ± SD, median (P25, P75) or number (%).

NRS: numerical rating scale; SD: standard deviation.

Intraoperative fentanyl consumption in group T was (0.384 ± 0.095) mg, lower than that in group C (0.465 ± 0.053) mg, and the difference was statistically significant (*P* < 0.05) (Table 2), including 3 cases of rescue analgesia (0.8%) in group T and 19 cases (5.5%) in group C.

**Figure 2:**
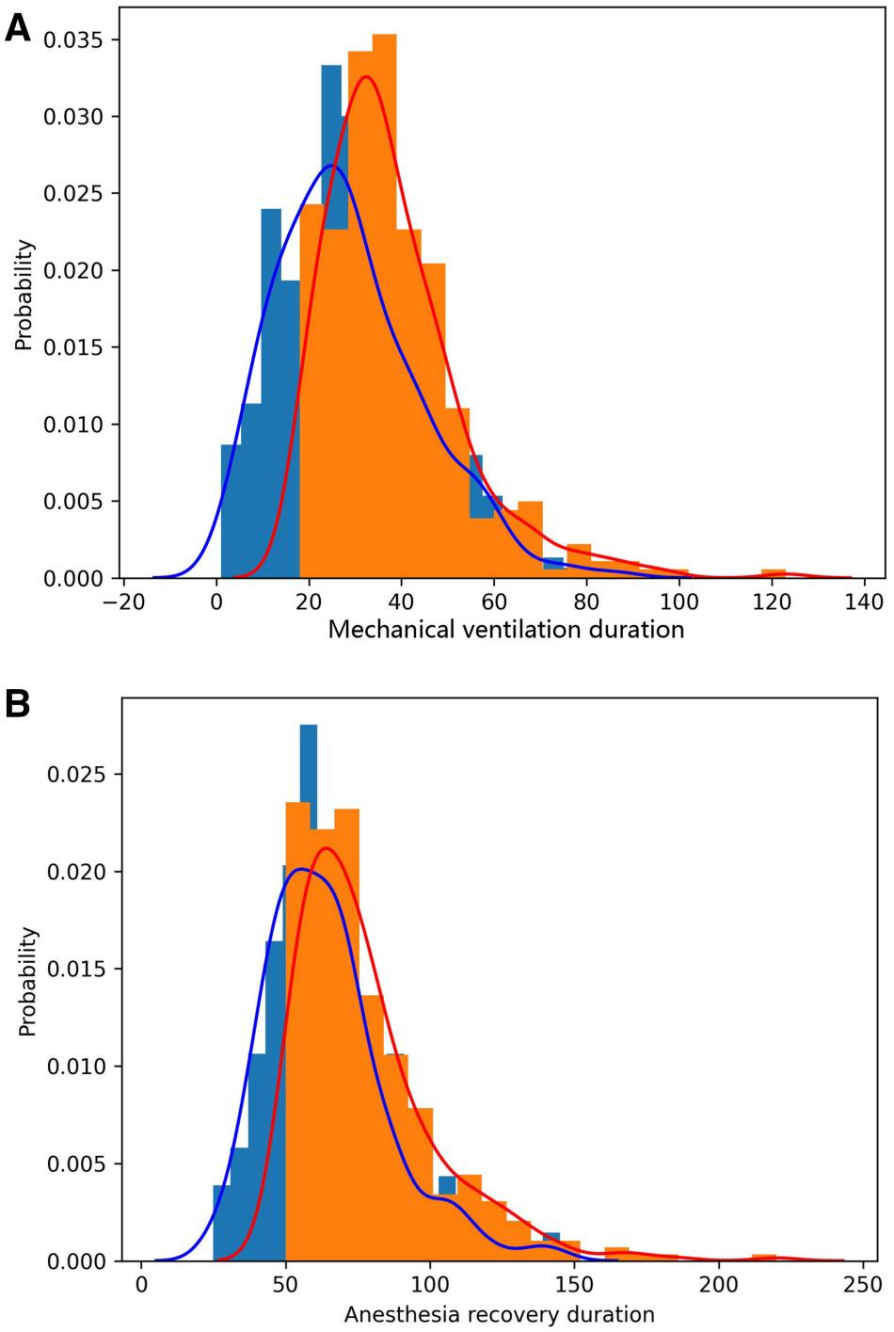
Frequency distribution histograms of mechanical ventilation and anaesthesia recovery duration (**A** and **B**, respectively).

The duration of mechanical ventilation, anaesthesia recovery and hospitalization in group T (28.47 ± 15.52 min, 64.01 ± 21.25 min, 4.48 ± 1.86 days) was significantly lower than group C (38.01 ± 14.99 min, 77.89 ± 24.69 min, 5.80 ± 2.15 days). There was a statistically significant difference between the 2 groups (*P* < 0.05) (Table 3). Figure 2 demonstrates a significant reduction in both the duration of mechanical ventilation and anaesthesia recovery for group T compared to group C. Additionally, the distribution of group T is more concentrated, while group C exhibits a higher propensity for extreme values.

**Table 3:** The duration of mechanical ventilation, anaesthesia recovery and hospitalization in the 2 groups.

Primary outcome variable	Group T (*n* = 345)	Group C (*n* = 345)	Statistics	*P*-value
Mechanical ventilation duration (min)	28.47 ± 15.52	38.01 ± 14.99	8.218	<0.001
Anesthesia recovery duration (min)	64.01 ± 21.25	77.89 ± 24.69	8.205	<0.001
Length of hospital stay (days)	4.48 ± 1.86	5.80 ± 2.15	8.679	<0.001

Values are represented as mean ± SD, median (P25, P75) or number (%).

SD: standard deviation.

### Secondary outcomes

Comparing the occurrence of adverse reactions at 24/48 h after surgery, such as nausea, vomiting, dizziness, pruritus and urinary retention. The incidence of urinary retention at 48 h after surgery was significantly lower in the T group (*P* < 0.05), respectively, while no significant difference was observed in other adverse reactions (Table 4).

**Table 4:** Adverse reactions at 24 and 48 h postoperation.

Secondary outcome variable	Group T (*n* = 345)	Group C (*n* = 345)	Statistics	*P*-value
Nausea and vomiting				
24 h	29 (8.4%)	20 (5.8%)	1.779	0.182
48 h	18 (5.2%)	11 (3.2%)	1.764	0.184
Dizziness				
24 h	37 (10.7%)	27 (7.8%)	1.722	0.189
48 h	23 (6.7%)	22 (6.4%)	0.024	0.877
Pruritus				
24 h	1 (0.3%)	5 (1.4%)	2.690	0.101
48 h	1 (0.3%)	5 (1.4%)	2.690	0.101
Urinary retention				
24 h	283 (82.0%)	297 (86.1%)	2.120	0.145
48 h	69 (20.0%)	132 (38.3%)	27.863	<0.001

Values are represented as mean ± SD, median (P25, P75) or number (%).

SD: standard deviation.

There were no significant differences between the 2 groups regarding the 24- and 48-h postoperative sedation scores and the total number of effective compressions of the analgesic pump within 48 h (Table 5).

**Table 5:** Sedation score, the patient satisfaction and total number of effective compressions of the analgesic pump.

Secondary outcome variable	Group T (*n* = 345)	Group C (*n* = 345)	Statistics	*P*-value
Sedation score				
24 h	0 (0, 0)	0 (0, 0)	0.578	0.563
48 h	0 (0, 0)	0 (0, 0)	0.000	1.000
Patient satisfaction, *n* (%)				
0	5 (1.4%)	39 (11.3%)	36.297	<0.001
1	98 (28.4%)	121 (35.1%)		
2	242 (70.1%)	185 (53.6%)		
PCIA compressions number				
3	3 (0, 10)	3 (0, 10)	0.138	0.890

Values are represented as mean ± SD, median (P25, P75) or number (%).

PCIA: patient controlled intravenous analgesia; SD: standard deviation.

The dissatisfaction rate in group T (1.4%) was lower than that in group C (11.3%), while the satisfaction rate (70.1%) was higher than that in group C (53.6%). Statistically significant differences were observed between the 2 groups (*P* < 0.05) (Table 5).

## DISCUSSION

Surgical resection remains the optimal treatment for patients with thoracic tumours [11]. However, due to the reduced immunity of tumour patients and the complex and traumatic nature of lobectomy, postoperative pain and pulmonary infection may ensue [12], ultimately diminishing the quality of survival. A prolonged hospital stay, respiratory complications and chronic post-thoracotomy pain syndrome can be associated with postoperative pain [13]. Numerous studies have substantiated that thoracic paravertebral block (TPVB) not only effectively ensures sufficient postoperative pain relief, diminishes opioid consumption and lowers the incidence of postoperative pulmonary infections but also expedites patients' prompt engagement in respiratory exercises, fosters recuperation of postoperative respiratory function, stabilizes patients' emotional well-being and enhances sleep quality [14, 15]. Ultrasound guidance allows for real-time visualization of the pleura, lung tissue and puncture needle, enhancing the success rate and safety of TPVB [14, 16]. Previous meta-analysis has demonstrated that TPVB offers analgesic efficacy that is on par with thoracic epidural analgesia, while also presenting a lower incidence of complications [17, 18].

Advanced analgesia is defined as an analgesic intervention administered before the emergence of harmful stimuli to prevent central sensitization [19], thereby reducing the incidence of hyperalgesia and allodynia [20]. A recent meta-analysis revealed that advanced analgesia significantly decreased postoperative pain scores, cumulative opioid consumption, postoperative nausea and vomiting and extended the time to first rescue analgesia [21]. To minimize the opioids in perioperative management to prevent the depression on respiratory function and cough reflexes, thereby facilitating the patient's respiratory recovery [22]. Furthermore, opioids induce immunosuppression by severely impairing innate immunity, altering antigen presentation and shifting the balance towards protumour cytokines [23, 24]. This effect leads to the growth and spread of residual tumour cells during the early postoperative period. Lee *et al.* [25] confirmed that paravertebral nerve block could significantly reduce opioid consumption and improve the overall survival rate of lung cancer patients.

With the advent and promotion of ERAS, identifying analgesic strategies that effectively accelerate postoperative recovery has become a clinical focal point. The innovation of this study lies in the integration of ERAS and advanced analgesia concepts, facilitated by anaesthesiologists, surgeons and nurses, to expedite postoperative recovery and improve patients' quality of life. In our study, patients received analgesic education and pain assessment on the day before surgery. The combined analgesia methods, including preoperative UG-TPVB, intraoperative use of opioids and non-steroidal analgesics and postoperative connection to an intravenous analgesia pump, provide further evidence that multimodal advanced analgesia management can not only achieve sufficient analgesia with low-dose opioids, reduce the number of cumulative rescue analgesia events and prolong the time to first postoperative rescue analgesia, but also facilitate early removal of the endotracheal tube, shorten the duration of anaesthesia recovery, enable patients to mobilize sooner and reduce the length of hospital stay, thereby achieving enhanced recovery. In addition, there is a need to enhance postoperative pain management and assessment. In the event that the pain score exceeded 4 points, it is imperative to promptly notify the surgeon in charge for appropriate treatment.

### Limitations

This study has some limitations. First, the optimization and comparison of the dose and mode of UG-TPVB were not conducted. Secondly, the effects of tumour size and scope of surgical resection on postoperative pain and recovery were not considered. These limitations will be addressed in future studies to further optimize postoperative analgesia and recovery outcomes for patients undergoing thoracoscopic lobectomy.

Future research could also investigate the long-term effects of advanced analgesia management on patients' quality of life and overall survival, as well as the potential impact on recurrence rates. Additionally, exploring the application of other analgesic techniques or pharmacological agents within the context of ERAS could help to identify more effective strategies for postoperative pain management and further improve patient outcomes. Moreover, a more comprehensive assessment of patient satisfaction and factors influencing their perception of pain could provide valuable insights for tailoring personalized pain management plans.

## CONCLUSION

In conclusion, the combination of ERAS principles and advanced analgesia concepts has shown promising results in enhancing postoperative recovery and improving patients' quality of life after thoracoscopic lobectomy. The use of ultrasound-guided thoracic paravertebral nerve block, in conjunction with multimodal advanced analgesia management, has demonstrated effective pain control while reducing opioid consumption and associated adverse effects. Further research is needed to address the limitations of this study and to explore other analgesic techniques or agents within the ERAS framework to optimize postoperative pain management and recovery outcomes for patients with thoracic tumours.

## Data Availability

All relevant data are within the manuscript and its Supporting Information files.

## ABBREVIATIONS

ERAS: Enhanced recovery after surgery
NRS: Numeric rating scale
NSCLC: Non-small-cell lung cancer
TPVB: Thoracic paravertebral block
UG-TPVB: Ultrasound-guided thoracic paravertebral block
VATS: Video-assisted thoracoscopic
